# A Bregman-Split-Based Compressive Sensing Method for Dynamic Harmonic Estimation

**DOI:** 10.3390/e24070988

**Published:** 2022-07-17

**Authors:** Aobing Chi, Chengbi Zeng, Yufu Guo, Hong Miao

**Affiliations:** College of Electrical Engineering, Sichuan University, Chengdu 610044, China; 2019141440225@stu.scu.edu.cn (A.C.); zengchengbi@scu.edu.cn (C.Z.); 2019141480020@stu.scu.edu.cn (Y.G.)

**Keywords:** phasor estimation, Taylor–Fourier multi-frequency (TFM), compressive sensing (CS), Bregman split, cross entropy

## Abstract

In order to overcome the spectral interference of the conventional Fourier transform in the International Electrotechnical Commission framework, this paper introduces a Bregman-split-based compressive sensing (BSCS) method to estimate the Taylor–Fourier coefficients in a multi-frequency dynamic phasor model. Considering the DDC component estimation, this paper transforms the phasor problem into a compressive sensing model based on the regularity and sparsity of the dynamic harmonic signal distribution. It then derives an optimized hybrid regularization algorithm with the Bregman split method to reconstruct the dynamic phasor estimation. The accuracy of the model was verified by using the cross entropy to measure the distribution differences of values. Composite tests derived from the dynamic phasor test conditions were then used to verify the potentialities of the BSCS method. Simulation results show that the algorithm can alleviate the impact of dynamic signals on phasor estimation and significantly improve the estimation accuracy, which provides a theoretical basis for P-class phasor measurement units (PMUs).

## 1. Introduction

As power grid monitoring technology advances and measurement devices, such as phasor measurement units (PMUs), are introduced into the power grid, dynamic monitoring of the power system has become crucial in ensuring the reliable operation and control of the power grid. In particular, harmonic and inter-harmonic information can be used to protect and monitor power systems, such as high-impedance fault identification [1,2], intelligent islanding detection [3,4], and harmonic state estimation [5,6]. Harmonic distortion affects the power quality and is not only technically but also economically harmful to the operation and maintenance of the grid. Apart from that, the continuous input of nonlinear loads, such as power electronic equipment, leads to the injection of a large number of harmonics into the grid, resulting in severe distortions in the voltage waveform and degraded power quality [7]. Therefore, the accurate estimation of harmonics and inter-harmonics has significant practical value in engineering.

To tackle the challenges of the new measurement environment of distribution networks, many improved or new PMU methods have been proposed, which fall into two main categories: model-based phasor measurement methods and discrete Fourier transform (DFT)-based methods. The first category expands the dynamic phasor approximation with different mathematical models, including the recursive least squares (RLS) method, the harmonic phasor estimator (HPE) method, and the autoregressive moving average (ARMA) method [8,9,10,11]. These methods can measure harmonic phasors but require a detailed set model order to yield desirable results.

The Taylor–Fourier Transform (TFT) [12,13], which can efficiently handle dynamic phasors, is also included in the first category. Further improvements can be made to iteratively estimate the actual fundamental frequency to boost the accuracy of TFT methods in the non-normative case [14]. However, to produce accurate results with such methods, the set model order must be close to the actual model with a high signal-to-noise ratio [15]. To cope with these difficulties, Chen et al. [16] employed a sinc interpolation function-based estimator (SIFE), which can adapt to different harmonic bandwidths and improve the accuracy of harmonic phasor estimation. However, in practical applications, many simulations are needed to select the appropriate parameters for each harmonic phasor model, increasing the harmonic estimation error.

The second category, DFT-based harmonic phasor estimation methods, can effectively alleviate the harmonic error issue present in the first category. W. Premerlani and R.K. Mai et al. [17,18] enhanced harmonic phasor estimation by correcting the estimation error through serial phasor estimation, which can be computed by DFT. DFT can be viewed as a bank of filters that carry the same frequency bandwidth to measure the harmonic phasors. Therefore, despite its simplicity and low computational complexity, it fails to adapt well to harmonic frequencies. Under frequency deviation and inter-harmonic interference conditions, the errors remain large.

Interpolation and window functions can be generally employed to diminish the errors under the above-mentioned frequency bias conditions. Interpolated DFT (IpDFT) is used in [19] to deal with static non-scalar conditions in harmonic phasor estimation. D. Petri et al. [20] extended IpDFT to include the phasor derivatives of the second-order expansion of the three DFT components near the fundamental frequency. However, their measurements are susceptible to decaying direct-current (DDC) components, which may produce phasor errors of up to 20% [21]. In addition, the window function, while also reducing the error under frequency bias conditions, will be significantly less effective in preventing external interference. D. Beleg et al. [22] exploited the longer observation interval filtering feature to reduce the interference. However, this capability also reduces the dynamics of the tracked signal because of the demand for shorter observation windows and larger bandwidths.

The above-mentioned estimation algorithms can measure harmonic phasors in most cases but fail to accurately estimate harmonic phasors in the presence of DDC component offsets. There are several methods available for estimating the harmonic phasor with DDC components. The DDC amplitude and time constant were estimated by integrating the fault current in [23] but are subject to noise and inter-harmonic interference [24]. In [25,26], a Taylor expansion model was used to approximate the DDC components, with high computational complexity [27]. Alternatively, the DDC components can be modeled as an exponential function [28], but this requires a large number of iterative calculations [29].

In recent years, research in machine learning has combined traditional phasor estimation methods with compressive sensing algorithms to address the effect of inter-harmonics on the estimator accuracy and reduce the computational burden of the phasor solution [30,31,32,33]. This method allows for the accurate recovery of a specific signal with fewer data points while maintaining relatively short observation intervals and resisting inter-harmonic interference, avoiding the impact of large amounts of redundant data on the computational speed. M. Bertocco et al. [31] investigated super-resolution discrete Fourier transform analysis based on compressive sensing (CS). The shorter sampling sequences can be applied to PMU and harmonic analysis. However, it is applicable to static phasor models only.

To handle the above-described challenges, the Taylor–Fourier multi-frequency (TFM) model in CS was introduced to describe the dynamic phasors in the proposed algorithm, where the Taylor–Fourier (TF) basis [32] is adopted and developed for the CS-based CSTFM algorithm [33]. The model can represent dynamic phasors at fundamental, harmonic, and inter-harmonic frequencies with a high degree of accuracy, but the complexity of the TFM model makes CSTFM computationally demanding. Generally, the CSTFM algorithm significantly exceeds the current harmonic phasor specifications in accuracy, which makes the CSTFM method a potential alternative for harmonic or inter-harmonic phasor analysis in distribution networks [34].

In summary, this paper takes the previous work and combines Bregman iterations with the TFM model described above and the compressive sensing method that has performed well in the field of harmonic detection. This innovative method aims to accurately estimate the harmonic, inter-harmonic, and DDC components in the TFM model. Firstly, establishing a dynamic signal model based on the Taylor–Fourier multi-frequency transform helps to simultaneously estimate the harmonic and inter-harmonic components and obtain the DDC components. Based on the regularity and sparsity of the dynamic signal distribution, the phasor problem is transformed into a compressive sensing problem by introducing an auxiliary signal. On the basis of the Bregman split method, the dynamic phasor estimation is then obtained by reconstructing the signal through the optimization of the hybrid regularization algorithm. The process also considers higher-order derivatives that allow for simultaneous phasor estimation, making the phasor estimation more accurate and efficient. The cross entropy can be used as a loss function to measure the similarity between two distributions. In this case, this paper verified the accuracy of the model by using the cross entropy to measure the difference in the probability distribution between the estimated and actual values. As a result, the proposed algorithm can meet the requirements of most international standards on PMUs. It also reduces the time variant of the harmonic components and its impact on dynamic phasor estimation in multi-frequency phasor analysis, which significantly improves the estimation accuracy. An innovative integration of Bregman iterations and compressive sensing theory is proposed to introduce model constraints into the conventional signal reconstruction objective function and to derive an algorithm for signal reconstruction with the proposed BSCS method.

## 2. DDC Component Estimation

For signals containing fundamental, dynamic harmonic, inter-harmonic, and DDC components of the power system, the multi-frequency dynamic signal model $x\left(t\right)$ can be represented by the sum of the sinusoidal components of the amplitude and phase given by

(1) $$x\left(t\right)=x_{0}\left(t\right)+x_{1}\left(t\right)=\lambda e^{-\frac{t}{\tau }}+\sum_{f_{h}\in Y_{h}}R_{h}\left(t\right)\cos\left[2\pi f_{h}t+\phi_{h}\left(t\right)\right]$$

where $x_{0}\left(t\right)$ is the DDC component of the signal, $x_{1}\left(t\right)$ is the fundamental and harmonic signal, λ and τ and $R_{h}\left(t\right)$ and $\phi_{h}\left(t\right)$ are the amplitude and time constant of the DDC component and the harmonic phasor, respectively, and $Y_{h}$ is a universal set of frequencies containing harmonic multiples of the actual power system frequency $f_{1}$ and possible inter-harmonic frequencies.

### 2.1. Multi-Frequency Dynamic Signal TFM Model

Each component of $x_{1}\left(t\right)$ in Equation (1) can be associated with a dynamic phasor defined as

(2) $$X_{h}\left(t\right)=\frac{R_{h}\left(t\right)}{\sqrt{2}}e^{j\phi_{h}\left(t\right)},\;\mathrm{for}\;f_{h}\in Y_{h}$$

Thus, $x_{1}\left(t\right)$ in Equation (1) can be rewritten as

(3) $$x_{1}\left(t\right)=\frac{1}{\sqrt{2}}\sum_{f_{h}\in Y_{h}}\left[X_{h}\left(t\right)e^{j2\pi f_{h}t}+X_{h}^{*}\left(t\right)e^{-j2\pi f_{h}t}\right]$$

On the basis of the prominent inertia characteristics of the power system, this can be approximated by the Taylor series as

(4) $$X_{h}\left(t\right)=X_{h}+t\cdot X_{h}^{\left(1\right)}+\;,\;\ldots \;,\;+\frac{t^{K}}{K!}\cdot X_{h}^{\left(K\right)}$$

where $X_{h}^{\left(k\right)}$ is the *k*th-order derivative of $X_{h}^{}$ and K is the Taylor expansion order. Integrating factors such as model accuracy and algorithm operations, K is taken as 3 in this paper, i.e.,

(5) $$\begin{array}{l} x_{1}\left(t\right)=\frac{1}{\sqrt{2}}\left(X_{h}+X_{h}^{\left(1\right)}t+X_{h}^{\left(2\right)}t^{2}+X_{h}^{3}t^{3}\right)e^{j2\pi f_{h}t}+\frac{1}{\sqrt{2}}\left(X_{h}^{*}+X_{h}^{\left(1\right)*}t+X_{h}^{\left(2\right)*}t^{2}+X_{h}^{\left(3\right)*}t^{3}\right)e^{-j2\pi f_{h}t} \end{array}$$

Therefore, the discrete expression for $x_{1}\left(t\right)$ is

(6) $$\begin{array}{cc} x_{1}\left(kT\right)= & \frac{1}{\sqrt{2}}\left[X_{h}+X_{h}^{\left(1\right)}kT+X_{h}^{\left(2\right)}{\left(kT\right)}^{2}\right.\left.+X_{h}^{3}{\left(kT\right)}^{3}\right]e^{j2\pi f_{h}t}\\ & +\frac{1}{\sqrt{2}}\left[X_{h}^{*}+X_{h}^{\left(1\right)*}\left(kT\right)\right.\left.+X_{h}^{\left(2\right)*}{\left(kT\right)}^{2}+X_{h}^{\left(3\right)*}{\left(kT\right)}^{3}\right]e^{-j2\pi f_{h}t} \end{array}$$

where * denotes the conjugate calculator and T is the sample interval.

Substituting Equation (2) into Equation (6) yields the Taylor expansion expression for the signal, which is then sampled at a sampling frequency $f_{s}$. Assume that $x_{1}\left[n\right]$ is a finite sequence of length samples, N is an even number, and −N/2≤n≤N/2−1. The sampling interval $\Delta T=1/f_{s}$. Thus, the time reference for the dynamic phasor calculation is located at n=0 in the sample record, and the discretized signal expression $x_{1}\left[n\right]$ is given by

(7) $$x_{1}\left[n\right]=\sum_{h}\sum_{k=0}^{K}\frac{{\left(nT\right)}^{k}}{k!}\left[\frac{X_{h}^{\left(k\right)}}{\sqrt{2}}e^{j2\pi f_{n}nT}+\frac{X_{h}^{*\left(k\right)}}{\sqrt{2}}e^{-j2\pi f_{n}nT}\right]=\Phi X_{h}^{}$$

where Φ is the coefficient of $\left[X_{h}^{\left(k\right)},\ldots ,X_{h}^{\left(k\right)}\right]$ in the original equation and denotes a matrix of size $N\times \left[Y\left(K+1\right)\right]$ that is a Taylor–Fourier basis matrix of exponential terms. To avoid confusion with the similar alphabetic phasors in Equation (2), assume that $r=X_{h}^{}$, as a column vector of length $Y\left(K+1\right)$, describes the set $X_{h}=[X_{h}^{\left(1\right)},\ldots ,X_{h}^{\left(k\right)}]$ when $h\in \left\{1,\ldots ,Y\right\}$, so that we obtain $x\left[n\right]=\Phi r$.

Although a TFM model was initially developed, if the model in Equation (7) is directly used to estimate $X_{h}\left(t\right)$ and the rate of frequency change, it will generate a considerable amount of computation and fail to meet the requirements of high reporting rates.

### 2.2. Estimation of DDC Components in the Multi-Frequency Dynamic Signal

Power system signals often contain DDC components, which are often ignored in the commonly used harmonic phasor detection methods because of their low content and the detection difficulties [35]. However, once the DDC components are biased, they can seriously interfere with the lower-order harmonic components of the original signal. In this case, the DDC components have a significant impact and cannot be ignored.

In this paper, the DDC components within a narrow time window are approximated as a dynamic, lower-frequency cosine component model [36], given as

(8) $$x_{0}\left(t\right)=\lambda e^{-\frac{t}{\tau }}\approx \sqrt{2}a_{0}\left(t\right)\cos\left(2\pi f_{DDC}t+\varphi_{0}\left(t\right)\right)\left(-T_{w}/2\leq t\leq T_{w}/2\right)$$

where $a_{0}\left(t\right)$ and $\varphi_{0}\left(t\right)$ are the amplitude and initial phasor of the DDC components of the model, respectively, $T_{w}$ is the length of the algorithm’s observation time window, and $f_{DDC}$ is a lower frequency.

Then, based on the frequency sample principle, the dynamic phasor corresponding to the cosine components of the DDC frequency is described as $p_{0}\left(t\right)=a_{0}\left(t\right)e^{j\varphi_{0}\left(t\right)}$. For a time-domain signal $p_{0}\left(t\right)$ with a finite amplitude, it can be parametrically modeled based on the sampling theorem in the frequency domain [37], which is given by

(9) $$p_{0}\left(t\right)\approx \sum_{k=0}^{K_{0}}p_{0,k}e^{j2\pi \left(k-\left\lceil \frac{K_{0}}{2}\right\rceil \right)\Delta f_{d}t}$$

where $p_{0,k}$ is the frequency sampling value of the phasor $p_{0}\left(t\right)$ at a frequency of $\left(k-\left\lceil \frac{K_{0}}{2}\right\rceil \right)\Delta f_{d}$, $\left\lceil \cdot \right\rceil$ is a downward rounding operator, $\Delta f_{d}$ is the frequency domain sampling interval, $K_{0}$ represents the number of frequency-domain samples used for the parametric modeling of $p_{0}\left(t\right)$, and −T/2≤t≤T/2. To achieve greater accuracy in the above model, it is generally required that $\Delta f_{d}$ be less than 1/T. After modeling $p_{0}\left(t\right)$, an approximate characterization of the dynamic DDC cosine components and the fundamental dynamic components can be achieved.

In this paper, $N_{w}$ is assumed to be an odd number such that the moment t=0 is centered in the observation window. The discrete form of the DDC components fitted in Equation (8) can be represented by Equation (10).

(10) $$x_{0}=\frac{\sqrt{2}}{2}\left[\psi_{0}\;\psi_{0}^{*}\right]\left[\begin{array}{c} p_{0}\\ p_{0}{}^{*} \end{array}\right]=\frac{\sqrt{2}}{2}\Psi_{0}P_{0}$$

where $x_{0}$ is a column vector with $N_{w}$ samples of the signal $x_{0}\left(t\right)$, $p_{0}$ is a row vector including $p_{0,k}$, and $\psi_{0}$ is a matrix with $N_{w}$ $\exp\left(j2\pi \left[\left(k-\left\lceil \frac{K_{0}}{2}\right\rceil \right)\Delta f_{d}+f_{DDC}\right]\right)$ sampled points in each column.

The least-squares method of Equation (10) provides the best parameters as it yields the minimum error between $P_{0}$ and the second-order Taylor approximation. At this point, it is the optimal solution subject to the following constraints

(11) $$\min{\left\|\Psi_{0}P_{0}-x_{0}\right\|}_{2}$$

where ${\left\|\cdot \right\|}_{2}$ denotes the Euclidean norm. Then, introducing the Lagrange multiplier and the Hermitian operator for derivation, the vector of coefficients of the phasor can be solved as

(12) $$P_{0}={\left(\Psi_{0}{}^{H}\Psi_{0}\right)}^{-1}\Psi_{0}{}^{H}x_{0}$$

where *H* denotes the Hermitian operator. In this way, according to Equations (9) and (12), the estimation of the DDC components can be obtained as

(13) $${\hat{p}}_{0}\left(t_{0}\right)\approx \sum_{k=0}^{K}{\hat{p}}_{0,k}$$

## 3. Harmonic Phasor Estimation

$r\in {\mathbb{R}}^{n}$ is the original signal, and we can obtain the observed data by calculating its *m* linear measurements. To make the formula more concise and easy to understand, $x\left[n\right]$ in Equation (7) is replaced here by A and is given by

(14) A=Φr

where Φ is the m×n matrix and $A\in {\mathbb{R}}^{m}$ is the observed value. The matrix Φ maps from ${\mathbb{R}}^{n}$ to ${\mathbb{R}}^{m}$, which represents the dimensionality reduction due to m≪n. Assume that the signal ***r*** can be compressed by the orthogonal transform Ψ and r=Ψβ. Then, (1) can be rewritten as

(15) A=ΦΨβ

where β is the conversion coefficient of the original signal ***r***, which can be transformed using a Gaussian random matrix Φ and a discrete wavelet inverse transform Ψ. The Gaussian matrix ΦΨ can satisfy the restricted isometry property condition with a high probability. Thus, when *m* is large enough, the coefficient β can be well reconstructed, and the original signal ***r*** can be solved by the inverse transformation r=Φβ. Once the signal ***r*** has been determined, we can calculate the harmonic’s frequency ${\hat{f}}_{h}$, amplitude ${\hat{X}}_{h}$, and frequency change rate ${\hat{R}}_{h}$ from [38].

### 3.1. Solving the Reconstruction Model

Power quality detection data are often contaminated by noise and spike anomalies. The anomalies are sparse and can be described with the $l_{1}$ parametrization. To obtain estimations with reasonable accuracy and robustness to harmonics and inter-harmonics, the estimation of ***r*** is represented as an optimization problem based on regularization, which means that the estimation results are converted to the original signal. The objective function of the reconstructed signal is given as

(16) $$T_{N}(\beta )=\frac{\mu }{2}{\left\|A-\Phi \Psi \beta \right\|}_{2}^{2}+{\left\|\beta \right\|}_{1}$$

where ${\left\|x\right\|}_{q}$ is the $l_{q}$ parametrization of any vector x whose $l_{q}$ parametrization is defined as ${\left\|x\right\|}_{q}={\left({\sum_{i=1}^{m}\left|x(i)\right|}^{q}\right)}^{1/q}$.

Many studies in this field have contributed to optimizing the objective function in Equation (16), most of which utilize the sparsity of the transform domain coefficients β. Because of the specific characteristics of the inter-harmonic signal, the regularization of Equation (16) allows for the introduction of an auxiliary signal ***s*** to obtain the new objective function T(β), which is given by

(17) $$T(\beta )=\frac{\mu }{2}{\left\|A-\Phi \Psi \beta \right\|}_{2}^{2}+{\left\|\beta \right\|}_{1}+{\left\|\frac{1}{\sqrt{\left|\nabla s\right|}}\nabla (\Psi \beta )\cdot \xi_{s}\right\|}_{2}^{2}$$

where

(18) $$\nabla (\Psi \beta )=\nabla r={\left[\begin{array}{c} r_{x}\\ r_{y} \end{array}\right]}^{T}$$

(19) $$\xi_{s}=\frac{1}{\left|\nabla s\right|}\left[\begin{array}{c} s_{y}\\ -s_{x} \end{array}\right]$$

∇ is the gradient operator, and $\xi_{s}$ is a unit vector perpendicular to the gradient of s. Ψβ is the signal ***r***; thus, ∇(Ψβ) is the gradient of ***r***, and $s_{x}$ and $s_{y}$ are the partial derivatives in the vertical and horizontal directions, respectively.

The new regularization takes the form of a dot production for the direction vector $\xi_{s}$ of the reference signal features and the gradient vector ∇(Ψβ) of the target signal. The proposed regularization penalty is weak if the edge direction of ***s*** and ***r*** is too small. Thus, we optimize the regularization expression ${\left\|(1\;\backslash \;\sqrt{\left|\nabla s\right|})\nabla (\Psi \beta )\cdot \xi_{s}\right\|}_{2}^{2}$ and obtain

(20) $${\left\|\frac{1}{\sqrt{\left|\nabla s\right|}}\nabla (\Psi \beta )\cdot \xi_{s}\right\|}_{2}^{2}={\left\|\frac{1}{{\left|\nabla s\right|}^{\frac{3}{2}}}\left[\begin{array}{cc} r_{x} & r_{y} \end{array}\right]\cdot \left[\begin{array}{c} s_{y}\\ -s_{x} \end{array}\right]\right\|}_{2}^{2}={\left\|\frac{1}{{\left|\nabla s\right|}^{\frac{3}{2}}}\left[\left(D_{x}\Psi \beta \right)s_{y}-(D_{y}\Psi \beta )s_{{}_{x}}\right]\right\|}_{2}^{2}$$

where $D_{x}$ is the difference operator in the x-direction and $D_{y}$ is the difference operator in the y-direction.

For the objective function (18), we introduce two regularization terms: one for the $l_{1}$ regularization of the coefficients β and another for the quadratic regularization that is constrained by the reference signal ***s***. ${\left\|(1\;\backslash \;\sqrt{\left|\nabla s\right|})\nabla (\Psi \beta )\cdot \xi_{s}\right\|}_{2}^{2}$ holds the edge direction of the signal ***r*** and ***s***. The term $\xi_{s}$ refers to the new regularization’s direction and $1\;\backslash \;\sqrt{\left|\nabla s\right|}$ controls its strength. If the gradient of ***s*** is slight while the projection ∇r in the $\xi_{s}$ direction is considerable, then a regularization penalty needs to be applied. Compressive sensing reconstruction can therefore be improved by hybrid regularization.

### 3.2. Bregman Split Method

The Bregman split method is essentially based on introducing an auxiliary variable to replace the problematic part of the original function, which simplifies the solution of the problem. In this paper, we solve the compressive sensing model by the Bregman split iteration to reduce the signal structure loss; thus, we call our proposed method the BSCS method. In the new function, based on the split criterion, we introduce auxiliary variables j and h. For these two constraints, μ is substituted by new regularization parameters σ and τ, which are employed to control the quadratic penalty function term. The expression is described as

(21) $$T(\beta )=\frac{1}{2}{\left\|A-\Phi \Psi \beta \right\|}_{2}^{2}+\frac{\sigma }{2}{\left\|j-\beta -h\right\|}_{2}^{2}+{\left\|j\right\|}_{1}+\frac{\tau }{2}{\left\|\frac{1}{{\left|\nabla s\right|}^{\frac{2}{3}}}\left[(D_{x}\Psi \beta )s_{y}-(D_{y}\Psi \beta )s_{x}\right]\right\|}_{2}^{2}$$

Based on the Bregman split iteration, the objective function of β can be computed efficiently. By breaking down the iterative process into several steps, we can reconstruct the signal by solving the following optimization:

(22) $$\beta^{k}=\arg\underset{\beta }{\min}\left\{\frac{1}{2}{\left\|A-\Phi \Psi \beta \right\|}_{2}^{2}+\frac{\sigma }{2}{\left\|j^{k}-\beta -h^{k}\right\|}_{2}^{2}\right.\left.+\frac{\tau }{2}{\left\|\left(1/{\left|\nabla s\right|}^{\frac{2}{3}}\right)\left[(D_{x}\Psi \beta )s_{y}-(D_{y}\Psi \beta )s_{x}\right]\right\|}_{2}^{2}\right\}$$

(23) $$j^{k}=\arg\underset{j}{\min}\left\{\frac{\sigma }{2}{\left\|j-\beta -h\right\|}_{2}^{2}+{\left\|j\right\|}_{1}\right\}$$

(24) $$h^{k}=h^{k}+\beta^{k}-j^{k}$$

There are two significant properties. First, during the iteration of Equation (22), ${\left\|A-\Phi \Psi \beta \right\|}_{2}^{2}$ decreases monotonically until it reaches zero. Furthermore, in the iterative solution of Equation (22), β monotonically converges to the true solution $\beta_{true}$ as long as ${\left\|A-\Phi \Psi \beta_{k}\right\|}_{2}^{2}>{\left\|A-\Phi \Psi \beta_{true}\right\|}_{2}^{2}$. Therefore, the Bregman method is stably convergent.

Regarding Equations (22)–(24), during each iteration cycle Equation (22) becomes a differentiable optimization problem. We can solve and update Equation (24) directly. Equation (23) can be solved efficiently with the definition of the shrink function to update the variable j, which is given by

(25) $$j^{k+1}=shrink(h^{k}+\beta^{k},1/\sigma )$$

where the shrink function is defined as

(26) $$shrink(x,1/\sigma )=\frac{x}{\left|x\right|}\cdot \max\left(\left|x\right|-\frac{1}{\sigma },0\right)$$

In Equation (22), Ψ, $D_{x}$, $D_{y}$, $s_{x}$, and $s_{y}$ can be considered constant matrices and vectors, and only β is variable. To minimize Equation (22), the first-order derivative concerning y is set to zero in the *k*th iteration. Before that, however, having first found the first-order derivative concerning β, we then obtain

(27) $$\begin{array}{l} \partial {\left\|\frac{1}{{\left|\nabla s\right|}^{\frac{3}{2}}}\left[(D_{x}\Psi \beta )s_{y}-(D_{y}\Psi \beta )s_{x}\right]\right\|}_{2}^{2}\\ =\frac{1}{2}\left(\frac{s_{y}}{{\left|\nabla s\right|}^{\frac{3}{2}}}\Psi^{T}D_{x}^{T}\frac{s_{y}}{{\left|\nabla s\right|}^{\frac{3}{2}}}D_{x}\Psi -\frac{s_{x}}{{\left|\nabla s\right|}^{\frac{3}{2}}}\Psi^{T}D_{y}^{T}\frac{s_{x}}{{\left|\nabla s\right|}^{\frac{3}{2}}}D_{y}\Psi \right.\\ \left.-\frac{s_{y}}{{\left|\nabla s\right|}^{\frac{3}{2}}}\Psi^{T}D_{x}^{T}\frac{s_{x}}{{\left|\nabla s\right|}^{\frac{3}{2}}}D_{y}\Psi +\frac{s_{x}}{{\left|\nabla s\right|}^{\frac{3}{2}}}\Psi^{T}D_{y}^{T}\frac{s_{y}}{{\left|\nabla s\right|}^{\frac{3}{2}}}D_{x}\Psi \right)\beta \end{array}$$

Following Equation (27), the linear regularized operator L is defined as

(28) $$\begin{array}{l} L=\left(\frac{s_{y}}{{\left|\nabla s\right|}^{\frac{3}{2}}}\Psi^{T}D_{x}^{T}\frac{s_{y}}{{\left|\nabla s\right|}^{\frac{3}{2}}}D_{x}\Psi -\frac{s_{x}}{{\left|\nabla s\right|}^{\frac{3}{2}}}\Psi^{T}D_{y}^{T}\frac{s_{x}}{{\left|\nabla s\right|}^{\frac{3}{2}}}D_{y}\Psi \right.\\ \left.-\frac{s_{y}}{{\left|\nabla s\right|}^{\frac{3}{2}}}\Psi^{T}D_{x}^{T}\frac{s_{x}}{{\left|\nabla s\right|}^{\frac{3}{2}}}D_{y}\Psi +\frac{s_{x}}{{\left|\nabla s\right|}^{\frac{3}{2}}}\Psi^{T}D_{y}^{T}\frac{s_{y}}{{\left|\nabla s\right|}^{\frac{3}{2}}}D_{x}\Psi \right) \end{array}$$

From L in Equation (28) and the first-order derivative of Equation (18), we obtain

(29) $${\left(\Phi \Psi \right)}^{T}\left(\Phi \Psi \beta^{k}-A\right)+\sigma \left(\beta^{k}-j^{k}+h^{k}\right)+\tau L\beta^{k}=0$$

By rearranging Equation (20) by $\beta^{k}$, a closed solution for $\beta^{k}$ is obtained as

(30) $$\left(\sigma I+\Psi^{T}\Phi^{T}\Phi \Psi +\tau L\right)\beta^{k}={\left(\Phi \Psi \right)}^{T}A+\sigma \left(j^{k}-h^{k}\right)$$

where I is an identity matrix. Since Equation (21) is linear, it can efficiently solve $\beta^{k}$. In the algorithm iteration of this paper, the cycles of Equations (23), (24), and (30) are combined and updated. When the regularization parameter τ = 0, the algorithm can calculate the coefficient β, which becomes the basic compressive sensing optimization.

After obtaining the coefficients β, the reconstructed phasor r is obtained by the equation r=Ψβ. Each of its rows corresponds to the phasor of each frequency component at a different time. An interpolation factor *F* is introduced to achieve more acceptable frequency-domain results. Additionally, $\Delta^{\prime }f=\Delta f/F$ is the frequency resolution. The reconstructed phasor frequency can be approximated as ${\hat{f}}_{h}≅l_{h}\Delta^{\prime }f$, where $l_{h}$ is the frequency index. Once ***r*** is determined, the frequency ${\hat{f}}_{h}$, the amplitude ${\hat{X}}_{h}$, and the rate of frequency change ${\hat{R}}_{h}$ of the required harmonic can be calculated by the following Equations (31)–(33)

(31) $${\hat{X}}_{h}=\sqrt{2}r_{h}^{\left(0\right)}$$

(32) f^h=l^hΔf+Imrh(1)/rh(0)2π

(33) R^h=Imrh(2)rh(0)−rh(1)2/rh(0)22π

where $r_{h}$ is the *h-*th column of ***r***, and $r_{h}^{\left(0\right)}$, $r_{h}^{\left(1\right)}$, and $r_{h}^{\left(2\right)}$ are the zero-order derivative, first-order derivative, and second-order derivative of $r_{h}$, respectively.

### 3.3. Cross Entropy

Cross entropy can be used as a loss function in machine learning. As cross entropy is a measure of the similarity between two distributions, the true distribution of the data set is p and the distribution corresponding to the outcome predicted by the model built is q. At this point, ‘cross entropy’ refers to the degree of difference between the predicted outcome q and the true outcome p. It is called the cross entropy loss function. The details are as follows.

Assume that the logistic regression model corresponding to the two categories has two zeros or ones. Given a prediction vector x, by means of the logistic regression function $g\left(z\right)=1/\left(1+e^{-z}\right)$, the true outcome y=1 corresponds to the predicted outcome $y^{\prime }=g\left(wx\right)$; the true outcome y=0 corresponds to the predicted outcome $y^{\prime }=1-g\left(wx\right)$.

The above is a description of the 0–1 distribution of the original data set through $g\left(wx\right)$ and $1-g\left(wx\right)$. From the definition of cross entropy, it follows that

(34) $$H\left(p,q\right)=-\sum_{i}p_{i}\log q_{i}=-y\log\hat{y}-\left(1-y\right)\log\left(1-\hat{y}\right)$$

The equation above is the cross entropy obtained for one sample of the test set. With the N samples in this paper, the corresponding cross entropy loss function is expressed as

(35) $$L\left(p,q\right)=\frac{1}{N}\sum_{n=1}^{N}H\left(p,q\right)=-\frac{1}{N}\sum_{n=1}^{N}\left[y\log\hat{y}+\left(1-y\right)\log\left(1-\hat{y}\right)\right]$$

We introduce the reconstructed phasor r to measure the difference in the probability distribution between the estimated value $\hat{r}$ and the theoretical value

(36) $$L\left(r,\hat{r}\right)=-\frac{1}{N}\sum_{n=1}^{N}\left[r\log\hat{r}+\left(1-r\right)\log\left(1-\hat{r}\right)\right]$$

Assuming a binary distribution of errors, L→0 can be considered a very close correlation between the predicted probability distribution and the actual probability distribution, which proves that the hypothetical model is consistent with the predicted model.

In summary, this paper takes the regularity of the dynamic harmonic frequency domain distribution as the optimization objective of dynamic phasor reconstruction and employs an iterative regularization model algorithm based on the Bregman split method to reconstruct the dynamic phasors containing the Taylor expansion coefficients of each harmonic. Finally, we use the cross entropy to measure the distribution difference between the estimated and theoretical values, which ensures that the model is accurate. The algorithm is shown in Table 1.

## 4. Simulation Performance Tests

In this section, we compare the accuracy of the estimation algorithm proposed in this paper with comparative algorithms in the literature under different test conditions and analyze the results. The comparison algorithms include the split Bregman iteration-based compressive sensing method, the Taylor–Fourier transform (TFT), the compressive sensing Taylor–Fourier multi-frequency method (CSTFM), IpDFT, and the O-splines FIR filter (OFF) algorithm. Test scenarios include basic performance tests, frequency deviation tests, harmonic oscillation tests, and interference anti-interference tests.

To evaluate the effectiveness of the different methods, the total vector error (TVE) of the IEEE measurement standard is introduced in order to describe the relative deviation between the theoretical and estimated phasor. The TVE is closely related to amplitude and phasor angle errors but cannot reflect the variation in one aspect alone. Therefore, this paper introduces two additional metrics (the frequency error (FE) and the ROCOF error (RFE) [39]) to comprehensively evaluate the effectiveness of the phasor estimation. The five comparison algorithms all use the same rectangular observation window with a fundamental frequency bandwidth of 1 Hz. The sampling window length was set to five frequency periods.

### 4.1. Basic Performance Tests

To verify the algorithm’s effectiveness when the signal frequency deviates, we assume that the sampling window is five periods long and the bandwidth of the fundamental frequency is 1 Hz. We constructed a signal model with fundamental and dynamic components as shown in the equation below.

(37) $$x(t)=\lambda e^{-t/\tau }+\cos(2\pi f_{1}t+\varphi_{1}(t))+\sum_{h=2}^{Y}0.1\cos(2\pi f_{h}t+\varphi_{h}(t))\;(t\geq 0)$$

where $f_{1}$ is the fundamental frequency, which was set to 50 Hz, and $\varphi_{1}(t)$ and $\varphi_{h}(t)$ represent the fundamental and harmonic phasor angles, respectively, which were taken to be any value in the range of (−π,π). λ and τ, the amplitude and time constant of the DDC components, were set to 0.6 and 0.04 s, respectively. The low-frequency band harmonic number h was assumed to be 2–13, and the sampling frequency was 5 kHz.

**Figure 1:**
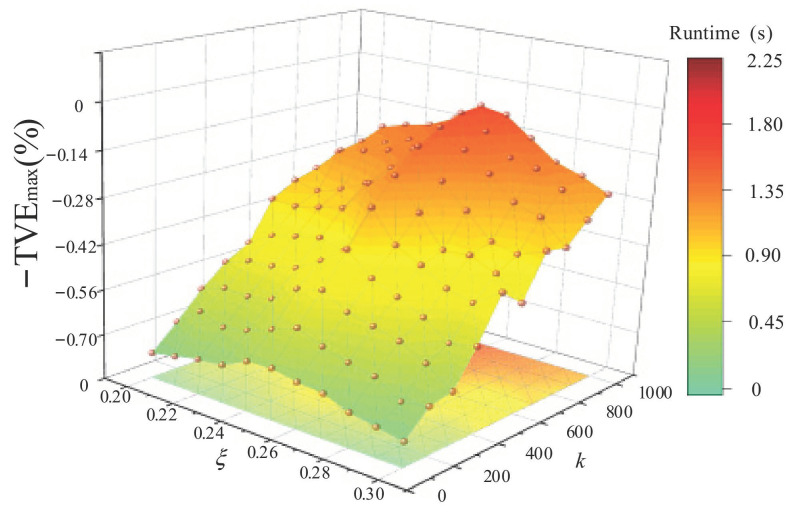
Reconstruction accuracy and runtime versus different parameters and iteration numbers.

When employing the algorithm, j and h were initialized as all-zero matrices and we set the regularization parameter μ=10. Since $\sigma =\xi \mu ,\tau =\left(0.5-\xi \right)\mu$, ξ is the balance parameter between parameters. The algorithm is more stable in the interval $\left[0.2,0.3\right]$, and *k* is the iteration number. Generally, the iteration number and the estimation accuracy are positively correlated within a specific range. The reconstruction effect and the algorithm runtime were analyzed while varying the parameters and the iteration number *k*. The results are shown in Figure 1.

As shown in Figure 1, as the iteration number *k* increases, the total phasor error gradually decreases. Still, the algorithm runtime continuously increases, and when *k* reaches 800, changing the iteration number has less of an impact on the accuracy, and the reconstruction accuracy becomes stable at this point. When the balance parameter ξ is varied between 0.2 and 0.3, the estimation accuracy first increases to the maximum value and then decreases, and its peak is around 0.25. Therefore, appropriately reducing the iteration number *k* and choosing a balance parameter can improve the reconstruction performance. Considering the reconstruction performance and estimation accuracy, we set parameter *k* = 800 and balance parameter ξ = 0.25.

Using OFF, CSTFM, TFT, and IpDFT as the comparison algorithms, the estimation results of TVE, FE, and RFE for the proposed algorithm and the comparison algorithms are shown in Table 2.

As shown in Table 2, the maximum values of the TVE, FE, and RFE of the BSCS algorithm are 0.691%, 0.057 Hz, and 2.181 Hz/s, respectively. According to the IEEE standard, the required TVE, FE, and RFE values are 1.5%, 0.06 Hz, and 2.3 Hz/s, respectively [40]. It can be seen that the proposed method can fully meet the IEEE measurement standard. The TVE, RFE, and FE values of the proposed BSCS algorithm are lower than those of the other algorithms. The BSCS method has a better detection ability for dynamic signals.

The OFF algorithm’s error estimation is close to the proposed method’s error estimation, but its TVE, FE, and RFE metrics still do not meet the IEEE measurement standard. The reason for this is that this method has large frequency errors due to the considerable noise in the spatial step reconstruction process. As for the TFT, CSTFM, and IpDFT methods, they all reconstruct the DDC components through the second-order Taylor model, whose inherent expansion order will produce specific errors in the reconstruction process; thus, the accuracy is not high. While the accuracy of the CSTFM method is second only to that of the OFF algorithm and the BSCS method near the higher harmonics but close to the accuracy of the TFT method near the lower harmonics, the maximum values of the TVE, FE, and RFE indicators are 7.418%, 1.736 Hz, and 25.211 Hz/s, respectively, which fail to meet the IEEE measurement standards [40]. The maximum phasor errors for the IpDFT and TFT methods are 10.456% and 8.872%, respectively, and the accuracy of the FE and RFE measurements is also less than desirable. Under dynamic conditions, the Fourier transform model cannot track the phasor changes in the observation window, leading to an incorrect phasor evaluation.

### 4.2. Frequency Deviation Tests

In the case that an out-of-step fault occurs in the power system, the voltage signal frequency will change continuously. The measurement accuracy of the phasor, frequency, and rate of frequency change of the PMU is essential to out-of-step detrending control. The IEEE specifies that the absolute frequency deviation of the power system should always be less than 0.5 Hz. Therefore, $f_{h}=1$ Hz yields good passband and stopband performance around each harmonic frequency. As previously mentioned, we considered harmonics up to the 13th order, with a sampling frequency of 10 kHz. Each test involved 1000 runs, where the fundamental and harmonic phasors were distributed as random numbers. Therefore, to assess the impact of the algorithms under frequency deviation conditions, the five algorithms described above were used as comparison algorithms, and the specific dynamic signals are shown below.

(38) $$x(t)=\lambda e^{-t/\tau }+1.5\cos(2\pi f_{1}t+\varphi_{1}(t))+0.15\cos(2\pi f_{h}t+\varphi_{h}(t))\;(t\geq 0)$$

where $f_{1}$ varies from 49.5 to 50.5 Hz in steps of 0.2 Hz. The amplitude λ and time constant τ of the DDC components were set to 0.6 and 0.04 s, respectively. In this case, we can see that the BSCS algorithm is always more accurate than the other four algorithms in terms of the harmonic phasor, frequency, and ROCOF estimation. This is because the model, which is based on the dynamic phasor’s higher-order derivatives, helps us obtain better passband and stopband performance, especially for higher-order harmonics.

Under these conditions, the maximum TVE, FE, and RFE of the BSCS method are 0.30%, 0.025 Hz, and 0.2 Hz/s, respectively. In the IEEE standard, they are thresholded at 1.5%, 0.01 Hz, and 0.4 Hz/s, respectively. Therefore, the BSCS method satisfies all the estimation requirements. The proposed estimation algorithm exhibits higher performance when the frequency deviation affects the signal waveform.

**Figure 2:**
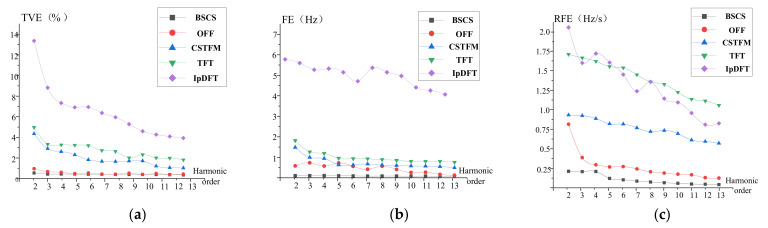
Estimation under frequency deviation conditions. (**a**) Maximum TVE; (**b**) maximum FE; (**c**) maximum RFE.

As shown in Figure 2, in terms of 2nd–13th-order harmonic estimation, none of the other methods meet the IEEE measurement standard. The IpDFT method has larger TVE, FE, and RFE values compared with the other methods and the lowest estimation accuracy and is rather sensitive to frequency deviation modulation. The estimation accuracy of the OFF, TFT, and CSTFM methods is better than that of the IpDFT method. The CSTFM method estimates the phasor based on a dynamic model, and the error values are less affected by frequency variations compared with the IpDFT method. Since the TFT method uses a second-order Taylor model to estimate the phasors, the estimation accuracy is not as high and is highly affected by frequency deviations. The OFF algorithm uses O-splines as the sampling operators to obtain the best Taylor–Fourier coefficients. It enables modulation at harmonic frequencies with an estimation accuracy close to that specified by the IEEE standard.

### 4.3. Harmonic Oscillation Tests

The signals used for the tests described in this section are as follows.

(39) $$\begin{array}{ll} x(t)= & 1.5\left[1+0.1\cos(2\pi f_{m}t)\right]\left[\cos(2\pi f_{0}t+0.1\cos(2\pi f_{m}t))+\phi_{1}\right]\\ & +\lambda e^{-t/\tau }+0.1\cos(2\pi hf_{0}t+\phi_{h})+0.1h\cos(2\pi hf_{m}t)\left(t>0\right) \end{array}$$

where $f_{m}$ is the modulation frequency, which was set to 5 Hz. The amplitude λ and time constant τ of the DDC components were set to 0.6 and 0.04 s, respectively. The sample rate was set to 5 kHz, and the sample period length was five cycles. The other parameters were the same as the values reported in the previous section.

**Figure 3:**
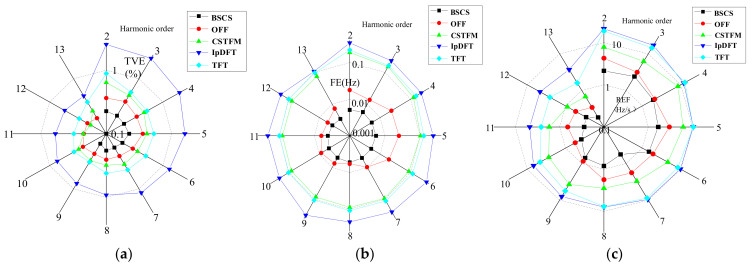
Estimation under harmonic oscillation conditions. (**a**) Maximum TVE; (**b**) maximum FE; (**c**) maximum RFE.

The estimation results graphically show only the parameter estimation results for the 2nd–13th-order harmonics. The estimation results are shown in Figure 3.

The harmonic oscillation has a more significant effect on the results of the BSCS algorithm at lower-order harmonics (e.g., 2nd–7th). However, the proposed algorithm is more accurate than the other methods in estimating higher harmonic parameters, especially the estimation of harmonic frequencies and the ROCOF. Under these conditions, the maximum values of the TVE, FE, and RFE for the BSCS method are 0.27%, 0.007 Hz, and 2.14 Hz/s, respectively, while those for the CSTFM method are 1.43%, 0.893 Hz, and 7.82 Hz/s, respectively. The maximum values of the TVE, FE, and RFE are respectively 1.49%, 0.924 Hz, and 18.74 Hz/s for the TFT method, 0.52%, 0.036 Hz, and 4.25 Hz/s for the OFF method, and 3.47%, 1.44 Hz, and 21.29 Hz/s for the IpDFT method. These data indicate that the TVE, FE, and RFE values of the proposed algorithm are the smallest, showing that the proposed method has a higher and more stable estimation accuracy under low-frequency oscillation conditions.

Under the test conditions described in Section 4.3, the corresponding thresholds in the IEEE standard for the TVE, FE, and RFE are 3.5%, 0.08 Hz, and 2.5 Hz/s, respectively, which are not satisfied by the four other comparison algorithms. The TFT method is subject to severe interference between adjacent harmonics in the case of harmonic oscillations, which affects the TFT method’s estimation accuracy. The OFF method provides an optimal algorithm for oscillation data compression, where the spline order controls the error. Therefore, the error’s impact is lower and close to that specified by the measurement standard. The CSTFM method introduces a TFM model to describe the dynamic phasors, but the model frequency cannot be selected accurately. This inaccurate signal model will lead to larger errors. The IpDFT method produces severe spectral leakage and inter-harmonic interference in the case of harmonic oscillations, so it is not suitable for harmonic frequencies with larger errors.

### 4.4. Anti-Interference Tests

**Figure 4:**
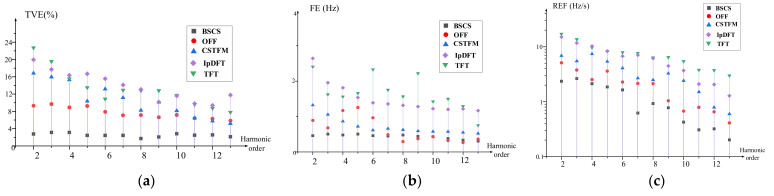
Estimation under inter-harmonic and noise interference conditions. (**a**) Maximum TVE; (**b**) maximum FE; (**c**) maximum RFE.

In general, power system signals contain inter-harmonics and a certain amount of noise, which seriously affect the estimation of the harmonic phasors. In the tests described in this section, Gaussian white noise with a signal-to-noise ratio of 60 dB was introduced to the signal. The dynamic signals were set as follows

(40) $$x(t)=\cos(2\pi f_{1}t)+0.1\cos(2\pi hf_{1}t)+\lambda e^{-t/\tau }+\mathrm{noise}+0.05\cos(2\pi f_{i}t)$$

where $f_{i}$ is the inter-harmonic frequency and h=2,…,13. The sampling rate was set to 5 kHz, and the sample period length was five cycles. The amplitude λ and time constant τ of the DDC components were set to 0.6 and 0.04 s, respectively. The simulation results are shown in Figure 4.

From Figure 4, we can see that the TVE, FE, and RFE values of the BSCS method are all smaller than those of the other algorithms. It can be concluded that the proposed algorithm has the highest estimation accuracy under dynamic conditions. Despite the local noise in the process, the reconstruction was almost complete.

The maximum TVE, FE, and RFE values for the BSCS method are 3.52%, 0.44 Hz, and 2.35 Hz/s, respectively, compared with 9.84%, 1.26 Hz, and 5.1 Hz/s for the OFF method, 22.97%, 2.65 Hz, and 14.93 Hz/s for the TFT method, and 22.36%, 2.65 Hz, and 14.98 Hz/s for the IpDFT method. Finally, the maximum TVE, FE, and RFE values for the CSTFM method are 17.42%, 1.32 Hz, and 6.88 Hz/s, respectively. According to the IEEE standard for the test conditions described in this section, the thresholds for the maximum TVE, FE, and RFE values are 3.4%, 0.45 Hz, and 2.5 Hz/s, respectively. Our proposed algorithm completely satisfies the standard requirements. The OFF algorithm has an optimal spatial sampler for bandlimited signals, provides an optimal data compression algorithm for oscillations in increasing degrees of splines, and delivers a powerful optimal state estimator that effectively suppresses noise and inter-harmonic interference. The TFT method is subject to severe interference between adjacent harmonics in the presence of noise interference; consequently, the estimation error of the TFT method is larger. In the case of the IpDFT algorithm, the inter-harmonic components in the vicinity of the fundamental harmonic cause a greater impact on the frequency estimation. Additionally, the estimation results can also be affected by the fundamental frequency offset. The proposed BSCS method reduces the time-variation in inter-harmonic components and their noise impact on dynamic phasor measurements in the multi-frequency phasor analysis, significantly improving the estimation accuracy.

## 5. Conclusions

This paper proposed a new dynamic phasor estimation method based on compressive sensing theory and the Bregman iteration method. In the proposed method, the DDC component is first estimated on the basis of the TFM model. The estimation models for harmonics and inter-harmonics are then developed based on compressive sensing, followed by the establishment of auxiliary variables and a solution by the Bregman method. Then, the dynamic phasor reconstruction problem is transformed into an optimization problem of a hybrid regularization algorithm to reconstruct the signal and estimate the dynamic harmonic phasor’s amplitude, frequency, and rate of frequency change. By using cross entropy, it was verified that the predicted probability distribution correlates very closely to the actual one, which proves the validity of the proposed model. The superiority of the proposed algorithm was verified through analytical calculations and simulations.

The performance of the proposed method was verified under different conditions, in which test signals such as frequency deviations, harmonic oscillations, and additional noise were employed to simulate severe operating environments. Under these conditions, the simulation data show that the proposed method’s TVE, FE, and RFE values satisfy most of the IEEE standards and that the errors are significantly minimized. The results of the simulations show that the proposed method can significantly improve the accuracy of dynamic phasor estimation compared with other popular methods. The proposed method focuses on achieving higher accuracy but increases the computational complexity and processing time. Moreover, if dense inter-harmonic components exist near the harmonic frequency, the proposed method may not provide satisfactory results. Thus, maintaining high accuracy under dense inter-harmonic conditions and achieving the rapid measurement of multi-frequency dynamic signals will be explored in future research.

## Figures and Tables

**Table 1:** A detailed description of the proposed method.

**(1). Initialization**
1. Input data *A*, the auxiliary signal ***s***
2. Initialize matrixes Φ , Ψ and parameters μ ,σ ,τ
3. Compress by the orthogonal transformation Ψ ,r←Ψβ
**(2). Regularization**
1. Regularize Equation (16) by s and obtain the objective function
2. Perform a quadratic optimization and obtain Equation (20)
3. Optimize by the Bregman splitting method
**(3). Iterative estimation**
1. Commence the Bregman split iteration in Equations (22) and (23),
2. Define the shrinkage equation to solve Equation (23)
3. Derive the partial derivative of Equation (20) and obtain *L* in Equation (28)
4. Obtain Equation (29) from Equation (28) and the first-order derivative of Equation (24), and then solve $\beta^{k}$ by Equations (29) and (30)
5. Cycle (23), (24), and (30), update to calculate the coefficient β, and then pass r=Ψβ to obtain the reconstructed phasor ***r***
6. Obtain ${\hat{X}}_{h}$, ${\hat{f}}_{h}$, ${\hat{R}}_{h}$ according to Equations (31)–(33)

**Table 2:** Comparison of the estimation accuracy among algorithms.

Index	*h*	BSCS	OFF	CSTFM	IpDFT	TFT
**TVE** **(%)**	2	0.691	2.28	7.418	10.456	8.872
	3	0.664	2.96	4.981	8.472	2.672
	4	0.576	2.74	4.653	8.714	2.739
	5	0.513	1.59	4.187	7.941	2.447
	6	0.428	1.44	3.588	7.036	2.387
	7	0.431	1.38	3.583	6.809	2.122
	8	0.417	0.77	3.122	6.247	1.891
	9	0.385	0.46	3.068	6.005	1.662
	10	0.393	0.27	3.072	5.311	1.523
	11	0.376	0.25	2.234	4.993	1.474
	12	0.382	0.28	1.882	4.434	1.430
	13	0.137	0.022	0.590	1.877	1.422
**FE** **(Hz)**	2	0.057	0.138	1.736	2.244	1.946
	3	0.056	0.136	1.357	2.127	0.702
	4	0.053	0.034	1.084	2.035	0.421
	5	0.056	0.051	0.873	2.176	0.318
	6	0.051	0.063	0.723	2.131	0.237
	7	0.050	0.090	0.792	2.309	0.154
	8	0.053	0.058	0.738	2.045	0.144
	9	0.049	0.064	0.691	1.976	0.141
	10	0.048	0.086	0.670	1.841	0.130
	11	0.042	0.054	0.665	1.943	0.127
	12	0.037	0.073	0.633	1.652	0.135
	13	0.037	0.022	0.590	1.877	0.138
**RFE** **(Hz/s)**	2	2.181	2.251	25.211	31.278	44.740
	3	1.392	2.138	17.482	27.072	13.662
	4	0.927	1.104	12.947	22.056	4.857
	5	0.685	0.993	4.087	12.361	2.562
	6	0.689	0.896	1.092	10.835	1.881
	7	0.564	0.784	0.702	8.569	1.198
	8	0.540	0.772	0.551	8.930	0.973
	9	0.503	0.665	0.483	7.034	0.802
	10	0.386	0.661	0.492	6.787	0.699
	11	0.279	0.757	0.474	5.725	0.604
	12	0.291	0.445	0.463	5.646	0.584
	13	0.283	0.342	0.452	4.724	0.591

## Data Availability

The data presented in this study are available on request from the corresponding author. The data are not publicly available due to Laboratory requirements.

## Nomenclature

TFM	Taylor–Fourier multi-frequency
CS	Compressive sensing
BSCS	Bregman split iteration-based compressive sensing
PMUs	Phasor measurement units
DFT	Discrete Fourier transform
IpDFT	Interpolated discrete Fourier transform
OFF	O-splines FIR filter algorithm
TVE	The total vector error
FE	The frequency error
RFE	The rate of change of frequency (ROCOF) error
$x_{0}\left(t\right)$	The DDC component
$x_{1}\left(t\right)$	The fundamental and harmonic signal
λ	The amplitude of the DDC component
τ	The time constant of the DDC component
$R_{h}\left(t\right)$	The amplitude of the harmonic phasor
$\phi_{h}\left(t\right)$	The time constant of the harmonic phasor
$f_{1}$	The actual power system frequency
$X_{h}^{}$	$\mathrm{Dynamic}\;\mathrm{phasor}\;\mathrm{associated}\;\mathrm{with}\;\mathrm{each}\;\mathrm{component}\;\mathrm{of}\;x_{1}\left(t\right)$
$X_{h}^{\left(k\right)}$	$\mathrm{The}\;k\mathrm{th}\;\mathrm{order}\;\mathrm{derivative}\;\mathrm{of}\;X_{h}^{}$
K	The Taylor expansion order
∗	The conjugate calculator
T	The sample interval
$x_{1}\left[n\right]$	The discretized signal expression
Φ	$\mathrm{The}\;\mathrm{coefficient}\;\mathrm{of}\;[X_{h}^{\left(k\right)},\ldots ,X_{h}^{\left(k\right)}]$ in the original equation
r	$\mathrm{exactly}\;\mathrm{equivalent}\;\mathrm{to}\;X_{h}^{}$
$a_{0}\left(t\right)$	The amplitude of the DDC components of the model
$\varphi_{0}\left(t\right)$	The initial phase of the DDC components of the model
$T_{w}$	The length of the algorithm’s observation time window
$p_{0}\left(t\right)$	The dynamic phasor corresponding to the cosine components of the DDC frequency
$\left\lceil \cdot \right\rceil$	The downward rounding operator
$\Delta f_{d}$	The frequency-domain sampling interval
$K_{0}$	The number of frequency-domain samples used for parametric modeling
$x_{0}$	$A\;\mathrm{column}\;\mathrm{vector}\;\mathrm{with}\;N_{w}$ $\;\mathrm{samples}\;\mathrm{of}\;\mathrm{the}\;\mathrm{signal}\;x_{0}\left(t\right)$
$p_{0}$	$A\;\mathrm{row}\;\mathrm{vector}\;\mathrm{including}\;p_{0,k}$
${\left\|\cdot \right\|}_{2}$	The Euclidean norm
*H*	The Hermitian operator
Ψ	The orthogonal transform
β	The conversion coefficient of the original signal
Φ	The Gaussian random matrix
$T_{N}(\beta )$	The objective function of the reconstructed signal
${\left\|x\right\|}_{q}$	The $l_{q}$ parametrization of any vector ***x***
***s***	An auxiliary signal for regularization
∇	The gradient operator
$\xi_{s}$	A unit vector perpendicular to the gradient of ***s***
$s_{x}$	The partial derivatives in the vertical direction
$s_{y}$	The partial derivatives in the horizontal direction
$D_{x}$	The difference operator in the ***x***-direction
$D_{y}$	The difference operator in the ***y***-direction
***j***, ***h***	The auxiliary variables based on the split criterion
***σ***, ***τ***	New regularization parameters to control the quadratic Penalty function term
shrink	The shrink function to update the variable
L	The linear regularized operator
I	An identity matrix
*F*	The interpolation factor
${\hat{f}}_{h}$	The frequency of the required harmonic
${\hat{X}}_{h}$	The amplitude of the required harmonic
${\hat{R}}_{h}$	The rate of frequency change of the required harmonic
$r_{h}$	The *h-*th column of ***r***
